# Thyroid hormones inhibit tumor progression and enhance the antitumor activity of lenvatinib in hepatocellular carcinoma via reprogramming glucose metabolism

**DOI:** 10.1038/s41420-025-02378-z

**Published:** 2025-03-08

**Authors:** Chun‑Cheng Yang, Yu-Chuan Yan, Guo‑Qiang Pan, Guang-Xiao Meng, Xiao Zhang, Lun-Jie Yan, Zi-Niu Ding, Dong-Xu Wang, Rui-Zhe Li, Guang-Zhen Li, Zhao‑Ru Dong, Tao Li

**Affiliations:** Department of General Surgery, Qilu Hospital, Shandong University, Jinan, China

**Keywords:** Cancer metabolism, Cancer genetics

## Abstract

Thyroid hormones (THs) dysfunctions have been demonstrated to be associated with the risk of developing different types of cancers. The role of THs in regulating hepatocellular carcinoma (HCC) progression is still controversial. We demonstrated that T3 can inhibit HCC progression by enhancing the expression of THRSP. Mechanistically, T3 can activate tumor suppressor LKB1/AMPK/Raptor signaling as well as oncogenic PI3K/Akt signaling in HCC. Interestingly, T3-induced THRSP can augment the activation of LKB1/AMPK/Raptor signaling, yet inhibit T3-induced PI3K/Akt signaling activation, thereby preventing mTOR-induced nuclear translocation of HIF-1α, and ultimately suppressing ENO2-induced glycolysis and HCC progression. More importantly, the exogenous T3 enhances the antitumor effect of multikinase inhibitor lenvatinib in vitro and in vivo by regulating glycolysis. Our findings reveal the role and mechanism of THs in HCC progression and glucose metabolism and provide new potential therapeutic strategies for HCC treatment and drug resistance reversal.

## Introduction

Metabolic reprogramming, a hallmark of cancer, encompasses alterations in glycolysis, glutamine addiction, and lipid metabolism, among other processes [1–3]. The Warburg effect, characterized by increased aerobic glycolysis, is widely recognized as a hallmark of this reprogramming [4]. Elevated glycolysis in cancer cells is critical for tumor metabolism, leading to enhanced glucose uptake, high lactic acid content of the metabolites, and a high ratio of glycolysis [5]. Disruptions in glycometabolism, particularly the shift from oxidative phosphorylation to glycolysis, are pivotal for sustaining the biosynthesis and energy needs of cancer cells, driving tumor initiation, angiogenesis, invasion, metastasis, immune evasion, and drug resistance [4, 6–8].

Hepatocellular carcinoma (HCC), the most prevalent primary liver malignancy, is associated with high morbidity and mortality globally [9]. Its insidious onset and lack of specific early symptoms make HCC the third leading cause of cancer-related deaths worldwide [10]. A key feature of HCC is its unique metabolic reprogramming which includes the most extensive alterations in glucose metabolism [11]. While normal liver cells continuously produce glucose through gluconeogenesis, HCC cells evade this process, relying on glycolysis through altered signaling pathways such as the LKB1/AMPK pathway [12–14]. Notably, drug-resistant HCC cells exhibit markedly elevated glucose uptake and lactate production, indicative of enhanced glycolysis [15]. This metabolic distinction between HCC and normal hepatocytes suggests that targeting these pathways could offer novel therapeutic approaches for HCC and overcoming drug resistance.

Thyroid hormones (THs), comprising primarily triiodothyronine (T3) and thyroxine (T4), exert effects through genomic and non-genomic mechanisms [16]. T4 is converted into the active form T3, which participates in various physiological and pathological processes, including cell growth, metabolism, tumorigenesis, and cancer progression [17–20]. The liver is a key target of THs, with THs playing significant roles in liver disease pathogenesis such as alcoholic liver disease and non-alcoholic steatohepatitis [21, 22]. While most studies indicate an inhibitory role for THs in HCC, with T3 modulating the T3/TRβ axis and switching the Warburg effect to oxidative metabolism [23–26], contradictory findings suggest that THs may also promote HCC angiogenesis and progression [27, 28].

Given the central role of glycolysis in HCC and the debated role of THs in regulating both HCC progression and glycolytic pathways, the effects and mechanisms of THs in HCC and glucose metabolism were investigated. Our study demonstrates that T3 inhibits HCC progression both in vitro and in vivo. Mechanistically, T3 induces THRSP, which enhances LKB1/AMPK/Raptor (regulatory-associated protein of TOR) signaling while inhibiting the excessive activation of the PI3K/Akt/mTOR pathway. This cascade suppresses ENO2-induced glycolysis and HCC progression. Furthermore, T3 potentiates the antitumor effects of the multikinase inhibitor lenvatinib, providing a promising strategy to enhance the therapeutic efficacy of lenvatinib in HCC.

## Results

### THs inhibit HCC progression by enhancing THRSP expression

**Fig. 1:**
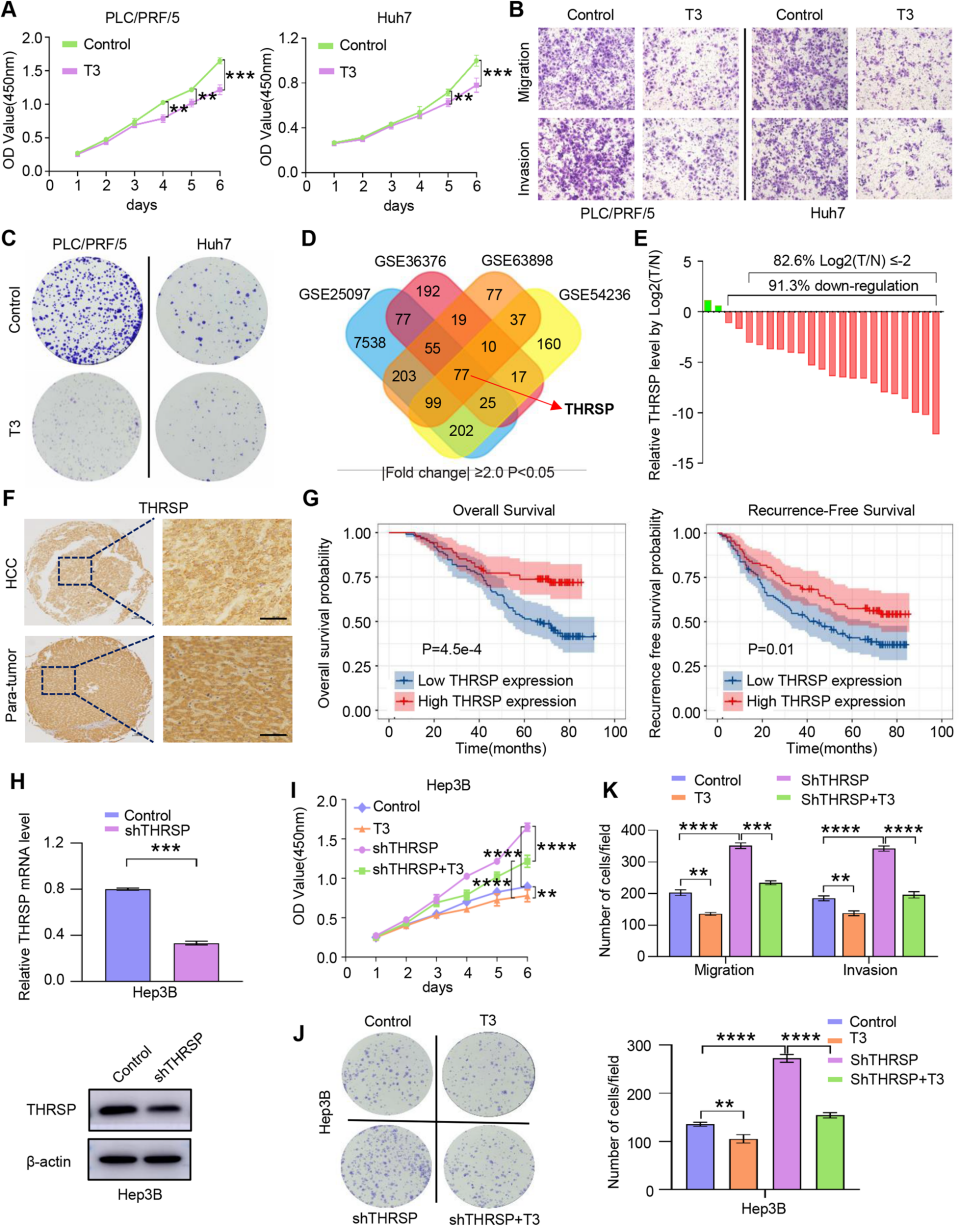
THs inhibit HCC progression by enhancing THRSP expression. **A**–**C** CCK-8, Transwell assay, and Colony formation assay were used to determine the influence of T3 on the proliferation, migration, invasion, and colony formation ability of HCC cells. **D** THRSP was significantly differentially expressed in HCC tissues from the GEO sequencing dataset. **E** qRT-PCR revealed that THRSP was significantly downregulated in HCC tissue samples. **F** The changes of THRSP expression in representative HCC samples in TMA were observed by IHC staining. Scale bar, 50 μm. **G** Kaplan-Meier analysis showed that the high THRSP expression in HCC tissues was associated with better OS and RFS. **H** qRT-PCR and WB confirmed the KD of THRSP in Hep3B cells. **I**–**K** THRSP knockdown attenuated the inhibitory effect of T3 on cell proliferation, clone formation, migration, and invasion ability in HCC cells. **P < 0.01; ***P < 0.001; ****P < 0.0001.

The effect of TH T3 on the phenotype of HCC cells in vitro was examined. Treatment with T3 (10 nM) significantly inhibited proliferation, migration, invasion, and colony formation in Huh7 and PLC/PRF/5 cells (Fig. 1A–C; Supplementary Fig. S1A, B). Subsequently, the molecular mechanisms underlying T3-mediated inhibition of HCC were investigated. THRSP, a key protein responsive to THs, is primarily expressed in tissues involved in fatty acid synthesis and lipid biosynthesis regulation [29]. Analysis of the GEO datasets revealed the reduced THRSP mRNA and protein levels in human HCC samples (Fig. 1D, E; Supplementary Fig. S1C, D). Western blotting and IHC on tissue microarrays confirmed the decreased THRSP protein expression in HCC, with THRSP levels emerging as an independent prognostic factor for both overall survival (OS) and recurrence-free survival (RFS) in patients with HCC (Fig. 1F, G; Supplementary Fig. S1E; Supplementary Tables S3, 4).

Evaluation of mRNA and protein expression across various HCC cell lines revealed a significant increase in THRSP expression 24 hours after T3 treatment (Supplementary Fig. S1F). To determine the role of THRSP in T3-induced HCC inhibition, Hep3B cells were used to establish THRSP knockdown (THRSP-KD) stable transfectants *via* lentiviral transduction, based on THRSP expression profiles (Fig. 1H, Supplementary Fig. S1G, H). Our results showed that THRSP knockdown reversed T3-mediated suppression of proliferation, colony formation, migration, and invasion in Hep3B cells which expressed higher THRSP expression (Supplementary Fig. 1I–K). These results suggest that T3 inhibits HCC progression through the regulation of THRSP expression.

### THRSP mediates THs-induced HCC inhibition and glucose metabolism by regulating LKB1/AMPK/Raptor and PI3K/Akt/mTOR pathway

**Fig. 2:**
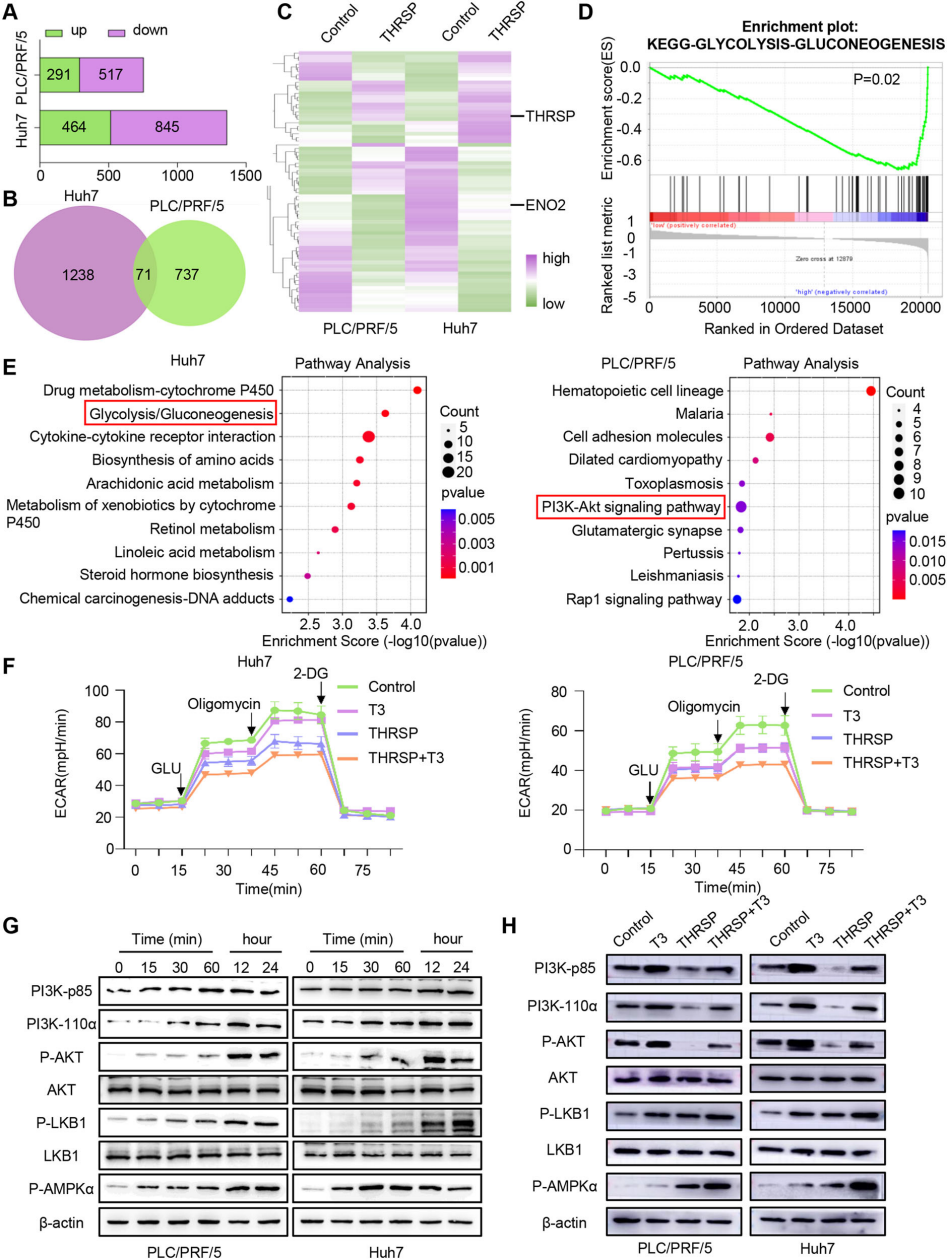
THRSP mediates THs-induced HCC inhibition and glucose metabolism by regulating LKB1/AMPK and PI3K/Akt pathway. **A**, **B** RNA-seq revealed the number of significantly up-regulated, downregulated, and common DEGs in THRSP-OE HCC cells. **C** Heat map of DEGs in THRSP-OE cells compared to the control group. **D** Genomic enrichment analysis of THRSP high and low expression in the TCGA database. **E** KEGG pathway enrichment analysis of the gene set negatively associated with THRSP in human HCC samples. **F** The effect of THRSP on glycolysis in T3-treated HCC cells. **G** T3 can activate the LKB1/AMPK signaling pathway and the PI3K/Akt signaling pathway in HCC. **H** WB showed that THRSP activated the LKB1/AMPK pathway, yet inhibited the PI3K/Akt signaling pathway in HCC.

The molecular mechanisms underlying T3 and THRSP-mediated suppression of HCC progression were subsequently investigated. First, Huh7 and PLC/PRF/5 cells, with low basal THRSP levels, were selected to generate THRSP overexpression (THRSP-OE) stable transfectants. Then, RNA sequencing (RNA-seq) of THRSP-OE and control (THRSP-Con) HCC cells revealed 1309 and 808 DEGs (|Fold change| ≥ 2, *P* < 0.05) in Huh7 and PLC/PRF/5 cells, respectively. A total of 71 common DEGs were identified, comprising 46 up-regulated and 25 downregulated genes (Fig. 2A–C; Supplementary Fig. S2A, B). Kyoto Encyclopedia of Genes and Genomes (KEGG) and Gene Ontology (GO) analyses highlighted significant involvement in the Glycolysis/Gluconeogenesis pathway, extracellular matrix composition, and transporter complexes (Fig. 2D; Supplementary Fig. S2C). Analysis of the TCGA database further confirmed the enrichment of THRSP-related genes in glycolytic regulation (Fig. 2E).

Glucose metabolism, a hallmark of metabolic reprogramming in cancer, not only supports accelerated tumor cell proliferation but also contributes to an acidic tumor microenvironment that facilitates invasion and immune evasion [30, 31]. Both THRSP-OE and exogenous T3 significantly reduced the glycolysis rate and glycolytic capacity in Huh7 and PLC/PRF/5 cells (Fig. 2F). Notably, THRSP-OE enhanced the inhibitory effect of T3 on glycolysis, whereas THRSP knockdown attenuated it (Fig. 2F; Supplementary Fig. S2D). These results suggest that THs regulate HCC glycolysis and progression through modulation of THRSP expression.

Next, potential signaling pathways involved in THRSP-mediated HCC inhibition were explored. The LKB1/AMPK pathway negatively regulates aerobic glycolysis in cancer cells, suppressing the initiation and progression of tumors including HCC [32, 33]. Conversely, the oncogenic PI3K/Akt pathway is often aberrantly activated in cancers, contributing to tumorigenesis and development [34]. Previous studies have demonstrated that T3 can activate LKB1/AMPK and PI3K/Akt signaling to influence hepatic metabolic processes and protect cardiomyocytes, respectively [35–37]. KEGG analysis of RNA-seq data from THRSP-OE and THRSP-Con HCC cells also revealed significant involvement of PI3K/Akt signaling (Fig. 2E). In vitro experiments confirmed that T3 activated both LKB1/AMPK and PI3K/Akt signaling in HCC cells, while THRSP-OE activated LKB1/AMPK signaling but inhibited PI3K/Akt signaling, and THRSP-KD inhibited LKB1/AMPK signaling but activated PI3K/Akt signaling (Fig. 2G, H; Supplementary Fig. S2E, F). These results suggest that THRSP mediates the TH-induced inhibition of HCC and glycolysis by regulating LKB1/AMPK and PI3K/Akt signaling pathways.

### THRSP mediates THs-induced HCC inhibition and glucose metabolism by inhibiting the transcription and expression of ENO2

**Fig. 3:**
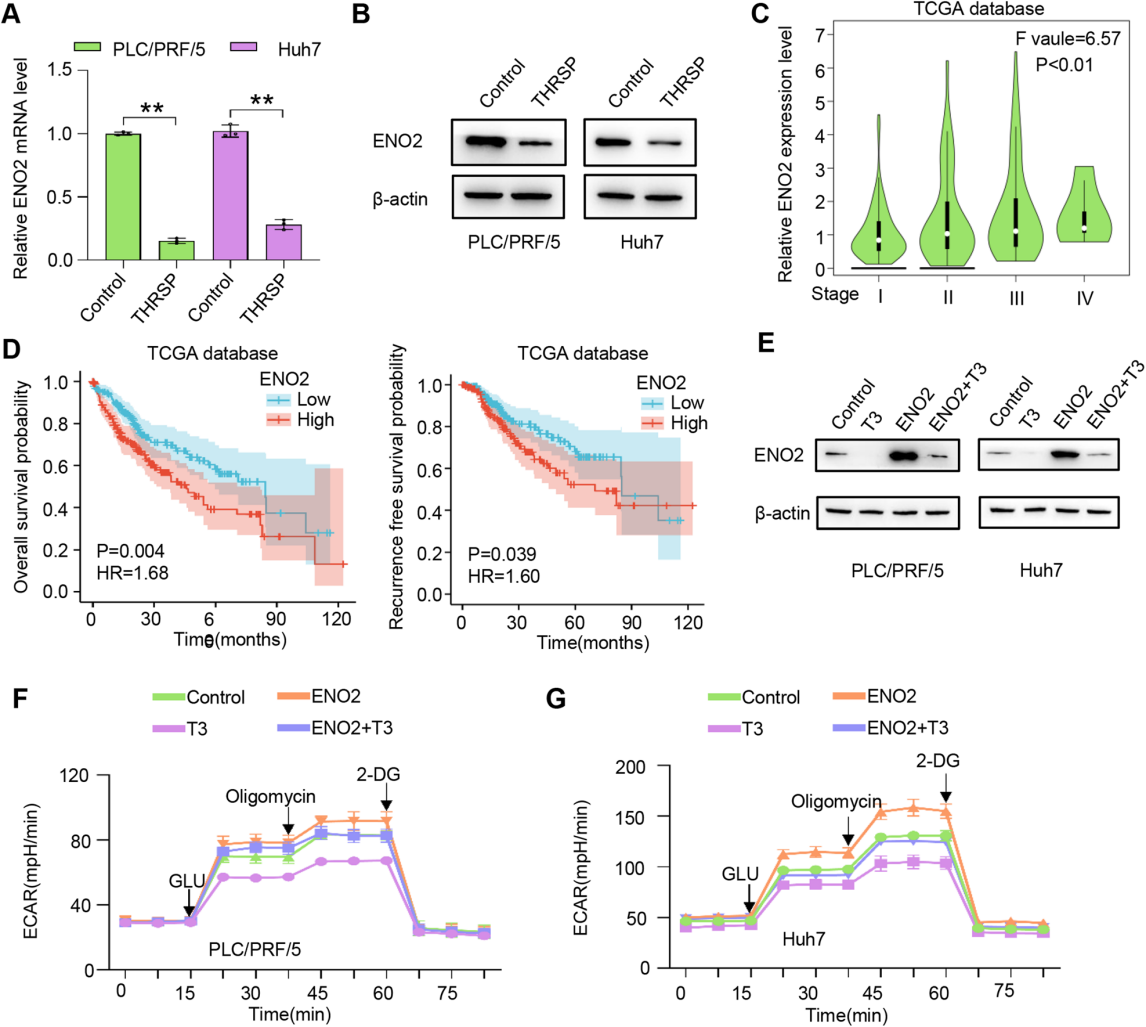
THRSP mediates THs-induced HCC inhibition and glucose metabolism by inhibiting the transcription and expression of ENO2. **A**, **B** qRT-PCR and WB confirmed that THRSP could regulate ENO2 expression in HCC cells. **C** The ENO2 expression was associated with the HCC tumor stage. **D** Kaplan–Meier analysis demonstrated that the ENO2 level was a predictive factor of OS and RFS in HCC patients. **E** WB results confirmed that T3 could inhibit the ENO2 expression in HCC in vitro. **F**, **G** ENO2 could attenuate the regulatory effects of T3 on glycolysis levels in HCC cells. **P < 0.01.

The downstream molecular mechanisms of THRSP in HCC progression were further elucidated. RNA-seq analysis revealed that, compared to the control group, ENO2 mRNA expression was downregulated in HRSP-OE cells, a finding confirmed by qRT-PCR and Western blot (Fig. 3A, B). TCGA database analysis indicated a correlation between ENO2 expression and tumor stage, OS, and RFS in HCC patients (Fig. 3C, D). ENO2, a rate-limiting enzyme in glycolysis, catalyzes the conversion of 2-phosphoglycerate (2-PG) to phosphoenolpyruvate, a critical step in the glycolytic pathway [38]. These findings led to the hypothesis that THRSP may mediate the T3-induced suppression of HCC progression and glucose metabolism by down-regulating ENO2 transcription and expression.

To test this hypothesis, in vitro experiments were conducted, showing that T3 treatment significantly inhibited ENO2 expression in PLC/PRF/5 and Huh7 cells (Fig. 3E). Stable ENO2-OE HCC cell lines were subsequently constructed, based on the basal ENO2 expression levels in different HCC cell lines (Supplementary Fig. S3A, B). Notably, ENO2 overexpression promoted cell proliferation, colony formation, migration, and invasion in HCC cells (Supplementary Fig. S3C–E), and also influenced glycolytic rate and glycolytic capacity (Fig. 3F, G). Furthermore, ENO2-OE reversed the inhibitory effects of T3 on glycolysis and the invasive behavior of HCC cells. These results suggest that T3 modulates glucose metabolism and HCC progression by inhibiting ENO2 expression.

### THRSP mediates THs-induced HCC inhibition through inhibiting mTOR-induced HIF-1α nuclear translocation

**Fig. 4:**
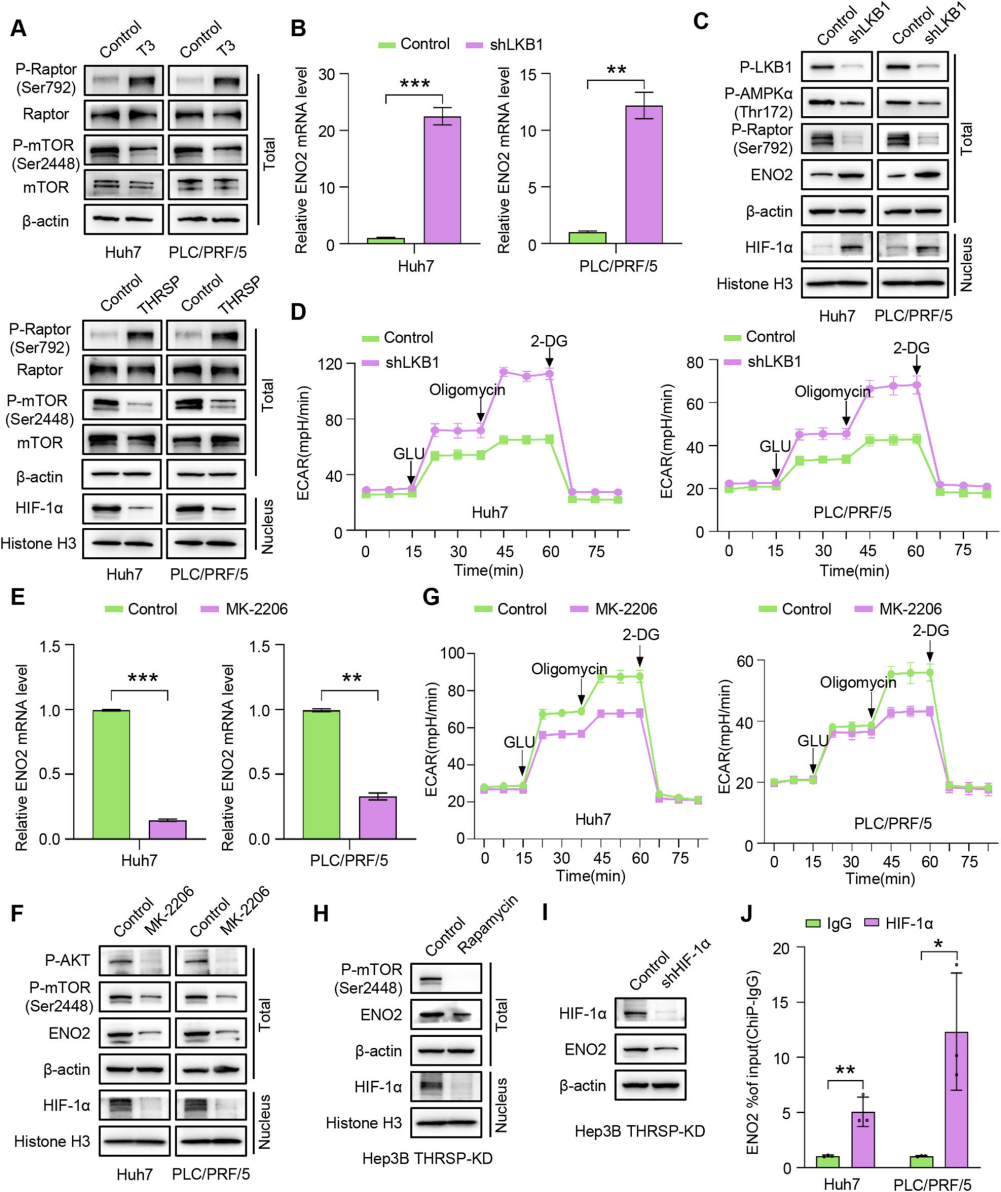
THRSP mediates THs-induced HCC inhibition through inhibiting mTOR-induced HIF-1α nuclear translocation. **A** T3 activates phosphorylation of Raptor and mTOR, while THRSP activates Raptor phosphorylation and inhibits mTOR phosphorylation and mTOR-induced HIF-1α nuclear translocation. **B**, **C** qRT-PCR and WB showed that LKB1 knockdown significantly inhibited the phosphorylation levels of AMPK and Raptor, and promoted nuclear translocation of HIF-1α and ENO2 expression. **D** LKB1 knockdown promoted glycolysis levels in HCC cells. **E** qRT-PCR detected the effect of MK-2206 treatment (10 μM, 48 h) on the ENO2 mRNA level in HCC cells. **F** WB analysis showed that MK-2206 reduced the levels of p-AKT, p-mTOR, and ENO2, and inhibited the nuclear translocation of HIF-1α in HCC cells. **G** MK-2206 reduces the glycolysis rate and glycolysis level in HCC cells. **H** Western blotting analysis was used to determine the effect of Rapamycin (10 μM, 48 h) on the levels of the indicated proteins in Hep3B THRSP-KD cells. **I** Knockdown of HIF-1α suppressed the ENO2 expression in Hep3B THRSP-KD cells. **J** ChIP-qRT-PCR showed statistically significant levels of HIF-1α bound to the ENO2 promoter in HCC cells. *P < 0.05; **P < 0.01; ***P < 0.001.

As a master regulator of cell growth and metabolism, the atypical serine/threonine kinase mTOR orchestrates anabolic processes and suppresses catabolic pathways by forming two structurally and functionally distinct complexes, mTORC1 and mTORC2 [39]. The mTOR pathway is interconnected with other signaling networks such as PI3K/AKT and AMPK, playing a pivotal role in cell growth regulation and cancer progression. AMPK can directly phosphorylate the Raptor component of mTORC1, thereby inhibiting mTORC1 activity and inducing cell-cycle arrest in response to energy stress [40, 41]. T3 promoted phosphorylation of Raptor at Ser792 and mTOR at Ser2448, while THRSP activated Raptor phosphorylation at Ser792 but inhibited mTOR phosphorylation at Ser2448 and HIF-1α nuclear translocation in HCC cells (Fig. 4A). These results suggest that THRSP may mediate T3-induced HCC inhibition and ENO2 downregulation by activating Raptor phosphorylation via the LKB1/AMPK pathway, which in turn suppresses mTOR activity and HIF-1α nuclear translocation in HCC.

Given the critical role of the LKB1/AMPK/Raptor axis in regulating mTORC1 kinase activity [42], the molecular mechanisms by which LKB1 inhibits mTOR-driven HIF-1α nuclear translocation were further investigated. Knockdown of LKB1 significantly reduced AMPK phosphorylation at Thr172 and Raptor phosphorylation at Ser792, while promoting HIF-1α nuclear translocation and ENO2 expression in THRSP-OE HCC cells (Fig. 4B, C). Additionally, LKB1 depletion enhanced glycolysis, proliferation, migration, and invasion in HCC cells (Fig. 4D; Supplementary Fig. S4A, B). To further confirm the essential role of T3-induced PI3K/AKT activation in mTOR phosphorylation at Ser2448 [43–45], the AKT inhibitor MK-2206 (10 µM) was used, which effectively inhibited mTOR activity, HIF-1α nuclear translocation, and ENO2 expression (Fig. 4E, F). This inhibition led to a reduction in glycolytic rate and capacity in HCC cells (Fig. 4G; Supplementary Fig. S4C, D).

Next, the role of mTOR and its downstream target HIF-1α in regulating ENO2 expression in THRSP-induced HCC cells was investigated. Treatment of THRSP-KD Hep3B cells with the mTOR inhibitor rapamycin suppressed HIF-1α nuclear translocation and ENO2 expression (Fig. 4H). Additionally, HIF-1α knockdown in THRSP-KD Hep3B cells resulted in reduced ENO2 expression (Fig. 4I). Finally, bioinformatics analysis using JASPAR software (http://jaspar.genereg.net/) indicated that the ENO2 promoter sequence may bind to transcription factor HIF-1α (Supplementary Tables S5). Chromatin immunoprecipitation (ChIP) assays confirmed that chromatin fragments from HCC cells immunoprecipitated with anti-HIF-1α antibody were significantly enriched compared to the IgG control group (Fig. 4J, Supplementary Tables S6).

### The ENO2 expression indicates a poor prognosis in HCC patients and is negatively correlated with THRSP level in vivo

**Fig. 5:**
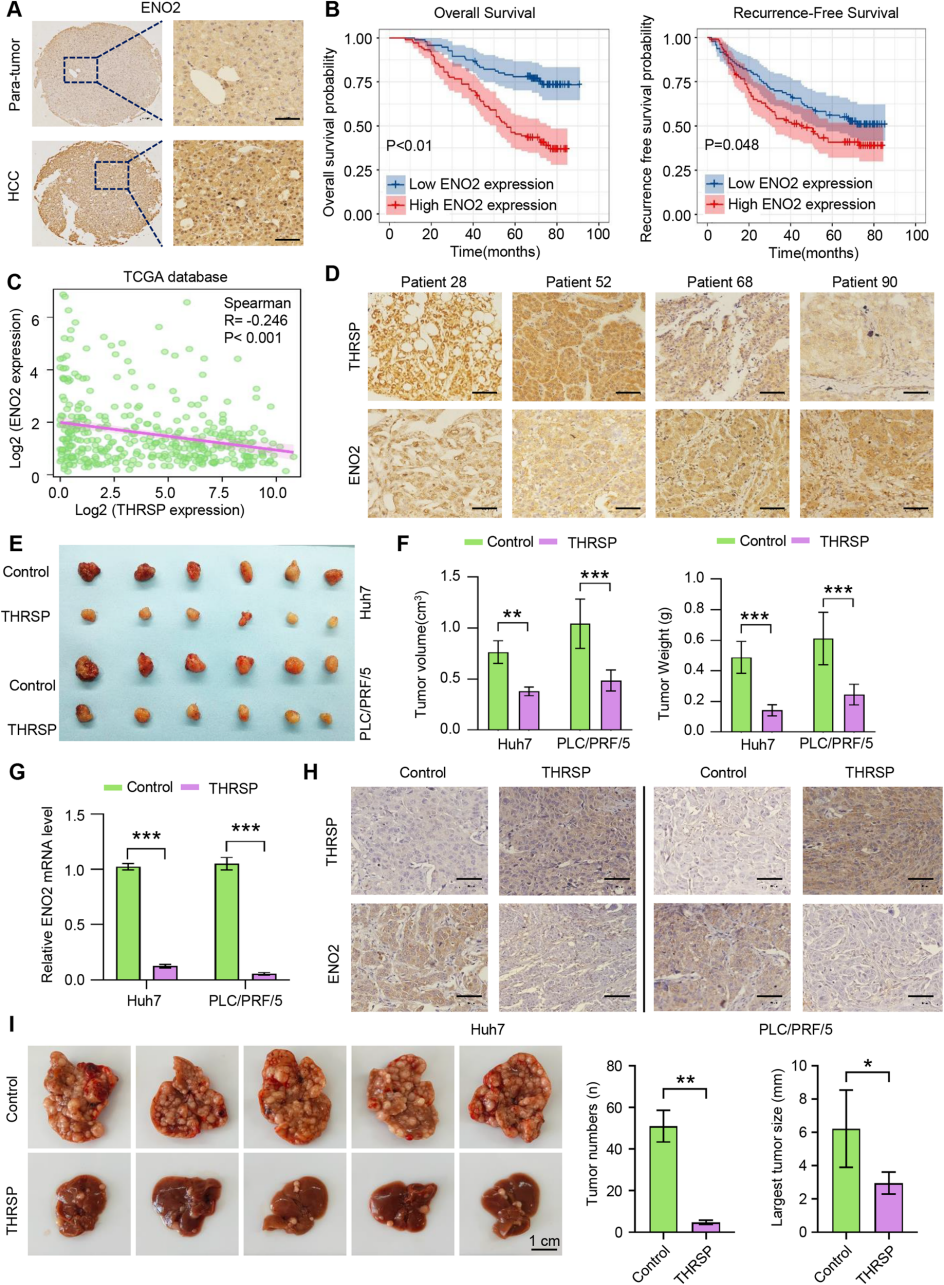
The ENO2 expression indicates a poor prognosis in HCC patients and is negatively correlated with THRSP level in vivo. **A** IHC results showed that ENO2 was upregulated in HCC samples. Scale bars, 50 μm. **B** Kaplan–Meier analysis showed that high ENO2 levels predicted worse OS and DFS in HCC samples. **C** THRSP expression was negatively correlated with ENO2 expression in the TCGA database. **D** IHC results showed that the THRSP expression was inversely correlated with ENO2 expression in HCC samples. Scale bars, 50 μm. **E**, **F** Xenograft tumors derived from Huh7-OE and PLC/PRF/5-OE cells were significantly smaller than those derived from the corresponding control cells. **G**, **H** qRT-PCR and IHC results confirmed the negative correlation between THRSP and ENO2 expression in Xenograft tumor tissue. **I** Overexpression of THRSP suppressed the number and size of tumors in the orthotopic HCC model. THRSP was over-expressed in vivo by utilizing adenovirus from intrahepatic injection. *P < 0.05; **P < 0.01; ***P < 0.001.

The role of THRSP in regulating ENO2 expression and HCC progression was further evaluated in vivo. Immunohistochemical analysis of HCC tissue microarrays revealed significant overexpression of ENO2 in human HCC samples compared to adjacent para-tumor tissues (Fig. 5A). Notably, patients with high ENO2 expression had poorer OS and RFS compared to those with low ENO2 expression (Fig. 5B). Multivariate analysis identified ENO2 expression as an independent risk factor for both OS and RFS in HCC patients (Supplementary Tables S7, 8).

Subsequently, the correlation between THRSP and ENO2 expression was analyzed in human HCC samples. In line with our functional findings, THRSP expression was found to be negatively correlated with ENO2 expression in the TCGA database (Fig. 5C), which was further validated by IHC analysis in our human HCC samples (Fig. 5D).

To assess the impact of THRSP on HCC progression in vivo, xenograft models of THRSP-Control and THRSP-OE HCC cells were established. Tumor volume and weight were significantly smaller in the THRSP-OE group compared to the control group (Fig. 5E, F). Moreover, *q*RT-PCR and IHC analysis confirmed an inverse correlation between THRSP and ENO2 expression in the HCC sample obtained from xenograft models (Fig. 5G, H). Finally, in an orthotopic HCC model, overexpression of THRSP led to a reduction in both the number and size of HCC tumors (Fig. 5I). These results collectively suggest that THRSP negatively regulates ENO2 expression in HCC and that ENO2 expression serves as a prognostic marker of poor outcomes in HCC patients.

### T3 synergistically enhances the antitumor activity of lenvatinib in HCC

**Fig. 6:**
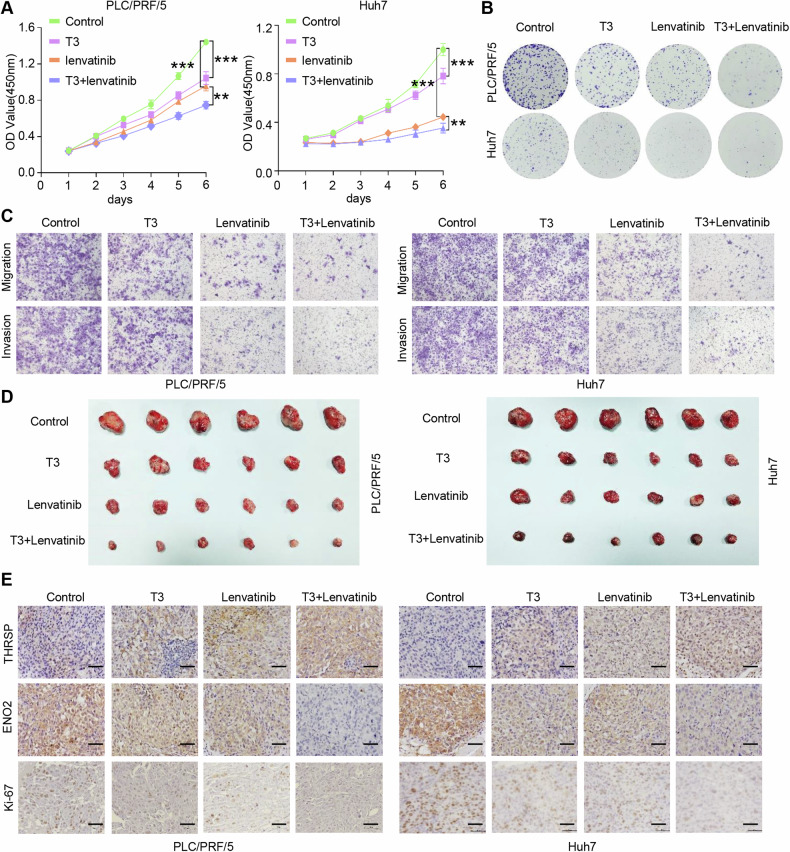
T3 synergistically enhances the antitumor activity of lenvatinib in HCC. **A**–**C** CCK-8, colony formation assay, and transwell assay showed the effects of T3 (10 nM) and lenvatinib (10 μM) treatment on HCC cell proliferation, colony formation, migration, and invasion. **D** Xenograft tumors that received combined therapy with T3 and lenvatinib showed better tumor growth inhibition. **E** IHC staining showed the levels of THRSP, ENO2, and Ki-67 in mouse xenograft tumors. Scale bars, 50 μm. **P < 0.01; ***P < 0.001.

Lenvatinib, a multi-targeted tyrosine kinase receptor inhibitor, is a first-line targeted therapy for advanced HCC [46]. Given that T3 could activate the AMPK/Raptor pathway, inhibiting the PI3K/Akt/mTOR-induced HIF-1α nuclear translocation, which has been implicated in lenvatinib resistance [47, 48], this study hypothesized a synergistic effect of T3 and lenvatinib in HCC treatment. In vitro experiments demonstrated that both T3 and lenvatinib alone inhibited HCC cell proliferation, colony formation, migration, and invasion. More importantly, T3 significantly enhanced the antitumor efficacy of lenvatinib in HCC (Fig. 6A–C; Supplementary Fig. S5A, B).

The efficacy of T3 and lenvatinib, both individually and in combination, on HCC growth and metastasis was further evaluated using Balb/c mouse HCC models. As shown in Fig. 6D, both T3 and lenvatinib alone effectively inhibited tumor growth, while the combination of T3 and lenvatinib significantly suppressed the tumorigenic capacity of HCC cells (Supplementary Fig. S5C). Additionally, the combination treatment markedly promoted THRSP expression and inhibited ENO2 and Ki-67 expression in HCC tumors in vivo (Fig. 6E). These results suggest that T3 can synergistically enhance the antitumor activity of lenvatinib in HCC by modulating ENO2-induced the process of glycolysis.

## Discussion

**Fig. 7:**
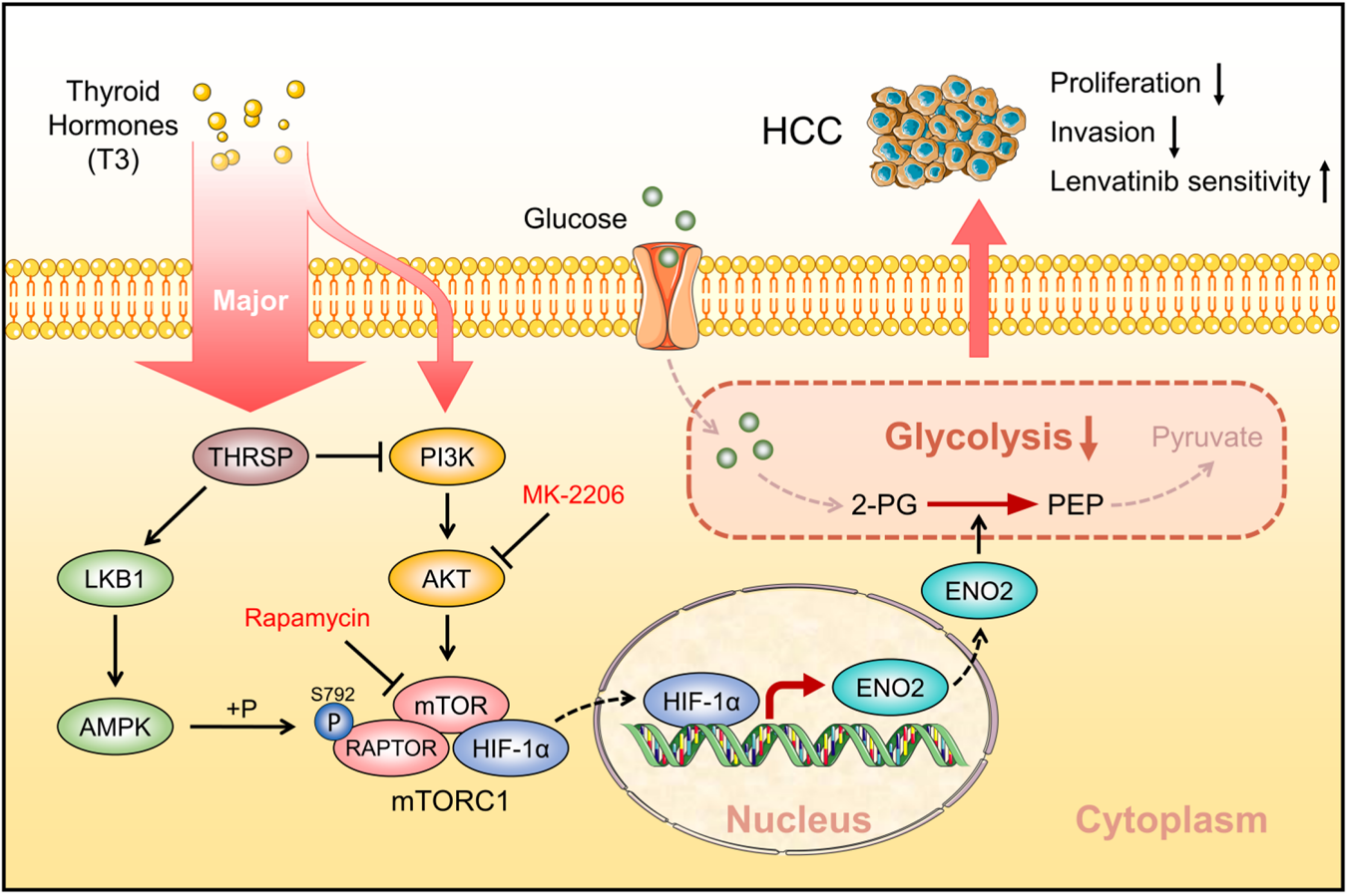
The schematic diagram showing THs inhibit HCC progression and reverse lenvatinib resistance through THRSP-mediated regulation of glycolysis.

Increasing evidence links TH dysfunction, including hyperthyroidism and hypothyroidism, to the risk of various cancers, highlighting the complexity of the molecular mechanisms regulated by THs [49]. Notably, the association between hypothyroidism and HCC has garnered particular attention. Although clinical studies suggest that hypothyroidism predisposes individuals to HCC development [50, 51], the precise role of THs in hepatocarcinogenesis and HCC progression remains contentious. This study affirms the antitumor effect of THs in HCC by inhibiting cell proliferation, colony formation, migration, and invasion both in vitro and in vivo. Additionally, THRSP is identified as a tumor suppressor whose expression is strongly correlated with the prognosis of HCC patients. Mechanistically, T3 activates the tumor suppressor LKB1/AMPK/Raptor pathway through the upregulation of THRSP expression, which concurrently inhibits T3-induced PI3K/Akt signaling, preventing mTOR-mediated nuclear translocation of HIF-1α. This cascade ultimately suppresses ENO2-driven glycolysis and HCC progression (Fig. 7). Furthermore, our findings demonstrate that T3 sensitizes HCC to lenvatinib by modulating glycolysis, providing novel insights into the role of THs in glycolysis regulation, drug resistance, and HCC progression.

As a regulator of fatty acid synthesis, THRSP is responsive to THs and is highly expressed in lipogenic tissues, including the liver, mammary glands, and adipose tissue [52]. In the liver, THRSP expression is rapidly induced by stimuli promoting long-chain fatty acid synthesis, such as THs, glucose, and insulin, and it plays a pivotal role in the pathogenesis of non-alcoholic fatty liver disease [29]. Despite its critical function in metabolism, the role of THRSP in tumorigenesis has been minimally explored, with conflicting results across different cancers [53, 54]. It is hypothesized that THRSP may execute divergent roles depending on the specific regulatory factors that induce its expression [52]. Our experiments confirm that THs can rapidly and persistently activate THRSP expression in human HCC cells [23]. The observation that THRSP inhibits glycolysis and HCC progression and serves as a prognostic marker for patient survival further underscores its potential as a therapeutic target for cancer treatment and glucose metabolism regulation in HCC.

Raptor, a key component of the mTORC1 complex, is essential for substrate recruitment and regulates mTORC1 activity [55, 56]. For instance, the deletion of REGγ enhances the stability of PP2Ac (protein phosphatase 2 catalytic subunit), stabilizing the interaction between PRAS40 and Raptor, thereby inactivating mTORC1-mediated hyperglycolytic metabolism and HIF-1α activation, which reduces DEN-induced liver tumor formation [57]. Additionally, AMPK activation can deactivate mTORC1 by directly phosphorylating Raptor at Ser722 and Ser792 [58]. Our study reveals that T3 activates the tumor suppressor LKB1/AMPK/Raptor signaling axis, ultimately suppressing ENO2-mediated glycolysis and inhibiting HCC progression.

As a key glycolytic enzyme, ENO2 plays a pivotal role in tumor glucose metabolism and has been implicated in enhancing cellular glycolysis and promoting tumorigenesis across various cancers, particularly in the conversion of 2-PG to phosphoenolpyruvate [38, 59]. ENO2 has also been shown to contribute to the progression of HCC [60]. Furthermore, ENO2 serves as a hypoxia-associated gene, influencing liver cancer development and prognosis [61]. However, its role in glycolysis has not been extensively studied. In contrast to previous studies, the present study performed KEGG pathway analysis on transcriptomic data and identified THRSP as a regulator of glycolytic processes. Further examination of glycolytic genes revealed significant alterations in ENO2 expression among the screened genes. Our findings underscore the essential role of ENO2 in HCC glycolysis, suggesting its potential as an effective therapeutic target for HCC.

Acquired resistance to therapy is a major contributor to cancer-related mortality, with metabolic reprogramming emerging as a key factor in therapy resistance [62, 63]. Cancer cells adapt to the tumor microenvironment by modifying metabolic pathways to sustain continuous growth, and an elevated glycolytic rate has been linked to increased resistance to treatment [64]. Recent studies suggest that glycolytic inhibitors are promising strategies to combat drug resistance in cancer [65]. However, cancer cells often alter their metabolic pathways to circumvent the effects of glucose metabolism inhibition [66]. To prevent such adaptations and resistance to glycolytic inhibitors, it may be beneficial to use these inhibitors in conjunction with existing therapies to enhance their efficacy. Moreover, given the widespread presence of glucose metabolism enzymes, including those involved in glycolysis, the systemic toxicity of glycolytic inhibitors could hinder their clinical application [67]. Our results, demonstrating that T3 sensitizes HCC to lenvatinib by modulating glycolysis, offer a promising and safer therapeutic approach for combination therapy in HCC.

## Conclusions

In summary, THs inhibit HCC progression and reverse lenvatinib resistance through THRSP-mediated regulation of glycolysis (Fig. 7). Our findings provide valuable insights into how reprogrammed glucose metabolism drives HCC progression, identifying key metabolic activities that could be leveraged for therapeutic targeting, and offering potential strategies for improving HCC treatment.

## Methods and materials

### Cell lines and cell culture

The human HCC cell lines PLC/PRF/5, Huh7, Hep3B, and MHCC97H were used in this study. These cells were cultivated in DMEM medium and MEM medium (BI; Bio Industries) supplemented with 10% fetal bovine serum (FBS; BI) and 1% penicillin-streptomycin (Solarbio). All cells were grown at 37 °C, 5% CO2, and a humidified environment.

### GEO datasets analysis

We selected the sequenced samples that were HCC and para-cancer tissue with a high number of samples in GEO databases. Screened genes that satisfied the criteria of |Fold change|≥ 2 and *P* value < 0.05 were identified as differentially expressed genes (DEGs).

### GEPIA analysis

The expression profiles of THRSP and ENO2 in HCC and normal tissues were obtained from the GEPIA website (http://gepia.cancer-pku.cn/), with additional data on ENO2 expression across different stages of HCC progression.

### Immunohistochemistry

For immunohistochemistry, paraffin-embedded tissue sections were first dewaxed in xylene and rehydrated through a graded ethanol solution. Antigen retrieval was achieved by heating the sections in citrate buffer (0.01 M, pH 9.0). Endogenous peroxidase activity was blocked by incubating with 3% hydrogen peroxide, followed by overnight incubation of the sections at 4 °C. After washing with phosphate-buffered saline (PBS), the slides were incubated sequentially with an enhancement solution and a secondary antibody (PV-9000; Zhongshan Biotechnology Co. Ltd.). Diaminobenzidine (DAB) was applied to visualize positive areas. Finally, the sections were dehydrated, mounted, and photographed, with subsequent image analysis performed.

### Tissue microarrays and survival analysis

These experiments were approved by the Ethics Committee of Qilu Hospital of Shandong University and were conducted in compliance with the approved guidelines. Immunohistochemical microarray data from 200 HCC clinicopathological cases and follow-up information were provided by Shanghai Outdo Biotech Company. This data was imported into SPSS 22.0 software for correlation analysis, and survival statistics were visualized using the Hiplot platform (https://hiplot-academic.com/?lang=zh_cn) to generate survival curves.

### Lentivirus transfection

Lentiviruses encoding human was purchased from GeneChem (Shanghai, China). The transduction process and establishment of stable cell lines were performed according to the manufacturer’s instructions.

### Real-time PCR

Total RNA from the cells was extracted using an RNA extraction kit (SparkJade, China) according to the manufacturer’s instructions. After determining the mRNA concentration, 10 µL of each sample was reverse transcribed into cDNA using Takara reverse transcription reagents. Quantitative real-time PCR (qRT-PCR) was conducted using SYBR Green (Genecopia). Relative mRNA expression was normalized to β-actin and calculated using the 2^−△△^Ct method. Primer sequences for the PCR of target genes are provided in Supplementary Table S1.

### Western blotting

Total proteins of tissues and cells were solubilized in a mixture of RIPA, protease inhibitor, and phosphatase inhibitor (Beyotime Institute of Biotechnology, 100:1:1). BCA protein analysis kit (Beyotime Institute of Biotechnology) was used to detect protein concentrations. Twenty mg of protein samples were separated by 10% SDS-PAGE and then transferred to PVDF membranes (EMD Millipore). Use 5% nonfat milk powder to cover for 1 hour, then put the membranes into the antibody against THRSP (1:1000; Proteintech Group), p-mTOR (1:1000; CST, Cell Signaling Technology), mTOR (1:1000; CST), PI3K-110α (1:1000; CST), PI3K-p85 (1:1000; CST), p-AKT (1:1000; CST), AKT (1:1000; CST), p-LKB1 (1:1000; CST), LKB1 (1:1000; CST), p-AMPK (1:1000; CST), AMPK (1:1000; CST), RAPTOR (1:1000; CST), ENO2 (1:1000; Abcam), HIF-1α (1:1000; Abcam), β-actin (1:3000; CST), Histone H3 (1:1000; Abcam) at 4 °C overnight.

### RNA sequencing and differential gene detection

THRSP overexpression and control lentiviruses were transfected into Huh7 and PLC/PRF/5 cells. RNA sequencing was performed by Annoroad Gene Technologies, with differential expression criteria set as |Fold change|≥ 2 and *P* value < 0.05 to identify DEGs.

### CCK-8 assay

Add 10 µl of CCK-8 reagent and 90 µl of blank medium to each well and incubate for an additional 1 hour in a cell incubator. Each group was equipped with 6 replicate samples, and absorbance was measured at 450 nm. In a 96-well plate, 1 × 10^3^ cells were spiked per space and measured every 24 h for 6 consecutive days to obtain OD values and plot cell proliferation curves.

### Cell migration and invasion assay

The bottom membrane of Transwell chambers (Corning Incorporate) was coated with 60 µl of Matrigel (1:8; Corning Incorporate) and left to settle at 37 °C for 1 hour. Then 200 µl of cell suspension of 5 × 10^4^ cells was added to the top of the chambers, and 700 µl containing 10% FBS was inserted underneath the chambers of the complete culture medium. After incubation for 36 h, the invading cells were fixed with methanol for 15 min and then stained with 0.1% crystal violet for 30 min. The number of invading cells was counted in five randomized views (×20).

### Colony formation assay

By cell counting, 1000 cells were grown in culture in six-well culture plates. After 10 days of complete medium culture, the colonies were fixed in methanol for 15 minutes, then stained with 0.1% crystalline violet for 30 min, and finally, the number of colonies was counted.

### In vivo tumor growth assay

All animal studies were conducted by the Guide for the Care and Use of Laboratory Animals established by the National Institutes of Health. HCC cells were counted at 2 × 10^6^ and injected subcutaneously into nude mice to establish a xenograft tumor model.

One week later, after most tumors reached 100 mm^3^ in volume, the mice were randomly divided into four groups based on tumor size, with six mice in each group: control group, T3 group, lenvatinib group, and T3 and lenvatinib combination group. Administration method: The T3 group was administered 45 ng/g by intraperitoneal injection; the lenvatinib group was administered 10 mg/kg by intraperitoneal injection, and the combined group was administered twice with an interval of half an hour; the control group was given an equal volume of PBS. Treatments were performed every three days, and all nude mice were euthanized after 5 weeks of treatment, and all tumors were completely removed.

For the orthotopic HCC model, pk330-sg-p53, pCMV-SB13, and pT3-EF1A-MYC vectors for hydrodynamic tail vein injection (HDTVi) were obtained from Biosune Company (Jinan, China). 2 ml sterile normal saline /plasmid mixture containing 10 ug each vectors above was injected into 6 weeks old C57BL/6 J mice. Orthotopic tumors were harvested 3 weeks after HDTVi.

### ChIP assay

ChIP was conducted using a Magna ChIP A/G kit (Millipore, Burlington, MA) according to the manufacturer’s instructions. ChIP quantitative real-time PCR was conducted as described. The sequences of the primers used for PCR are listed in Supplementary Table S2.

### Extracellular acidification rate

The Seahorse XFe 96 extracellular flux analyzer (Seahorse Bioscience) was employed to measure the extracellular acidification rate (ECAR) as an indicator of glycolytic activity. HCC cells (1 × 10^4^ cells/well) were plated in Seahorse cell culture plates, following the manufacturer’s protocol. After measuring basal ECAR levels, glucose, oligomycin, and 2-DG were added sequentially to each well at the specified time points to assess glycolytic activity. Data were analyzed using Seahorse XF-96 Wave software and expressed in mpH/min.

### Statistics analysis

RNA expression profiles and corresponding clinical information for patients with HCC were retrieved from the TCGA database (https://portal.gdc.com) and the GTEx database (https://gtexportal.org/). Statistical analysis was performed using the R programming language (v4.0.3), with graphical visualizations generated *via* ggplot2 (v3.3.2). A *P* value < 0.05 was considered statistically significant. All experiments were conducted independently three times, and statistical analyses of results were performed using GraphPad Prism 10 and IBM SPSS Statistics 22.0 software. OS and RFS were compared using Kaplan-Meier curves and log-rank tests. Multivariate Cox proportional hazards regression analysis was employed to identify independent prognostic factors. All tests were two-tailed, with p-values < 0.05 regarded as statistically significant.

## Supplementary information

Supplemental Material

Gel

## Data Availability

All data generated or analyzed in this study are included in this document or the supplementary materials, methods, tables, graphs, and or in the supplementary materials and methods, tables, figures, and legends document.
