# Supplementary material for: Thyroid hormones inhibit tumor progression and enhance the antitumor activity of lenvatinib in hepatocellular carcinoma via reprogramming glucose metabolism
Source: Cell Death Discov. 2025 Mar 8;11:92. doi: 10.1038/s41420-025-02378-z (PMC11889155; doi:10.1038/s41420-025-02378-z)
Supplement: Supplementary file 1 — Supplemental Material [file 41420_2025_2378_MOESM1_ESM.docx]

**Supplementary Figure and Figure Legends:**

**Figure S1** **THs inhibit HCC progression by enhancing THRSP expression**


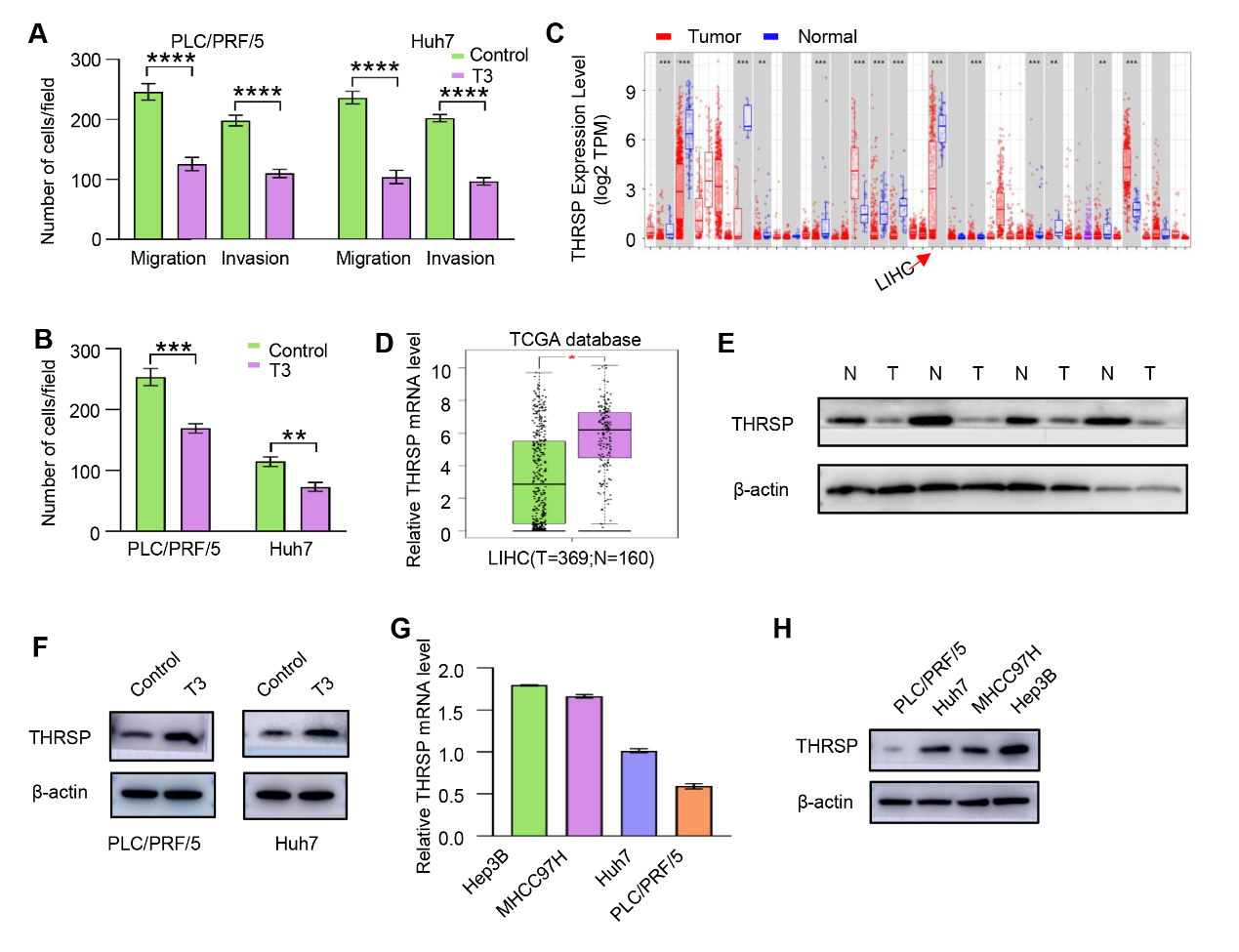
 (A-B) The inhibitory effect of T3 on the migration, invasion, and colony formation ability of HCC cells. (C) The TCGA database showed that THRSP mRNA was down-regulated in several cancers including HCC, compared to the corresponding normal tissues. (D) TCGA and GTEx databases revealed that the THRSP mRNA level was lower in tumor tissues than in normal liver tissues. (E) Western blot showed that the THRSP protein level was lower in HCC tissues than in normal tissues. (F) Western blot showed that the THRSP expression was increased after 24 hours of T3 treatment on HCC cells. (G-H) The THRSP expression in four HCC cell lines was detected by qRT-PCR and WB analyses. *, P < 0.05; ***, P < 0.001; ****, P < 0.0001.

**Figure S2 THRSP mediates THs-induced HCC inhibition and glucose metabolism by regulating LKB1/AMPK/Raptor and PI3K/Akt/mTOR pathway**


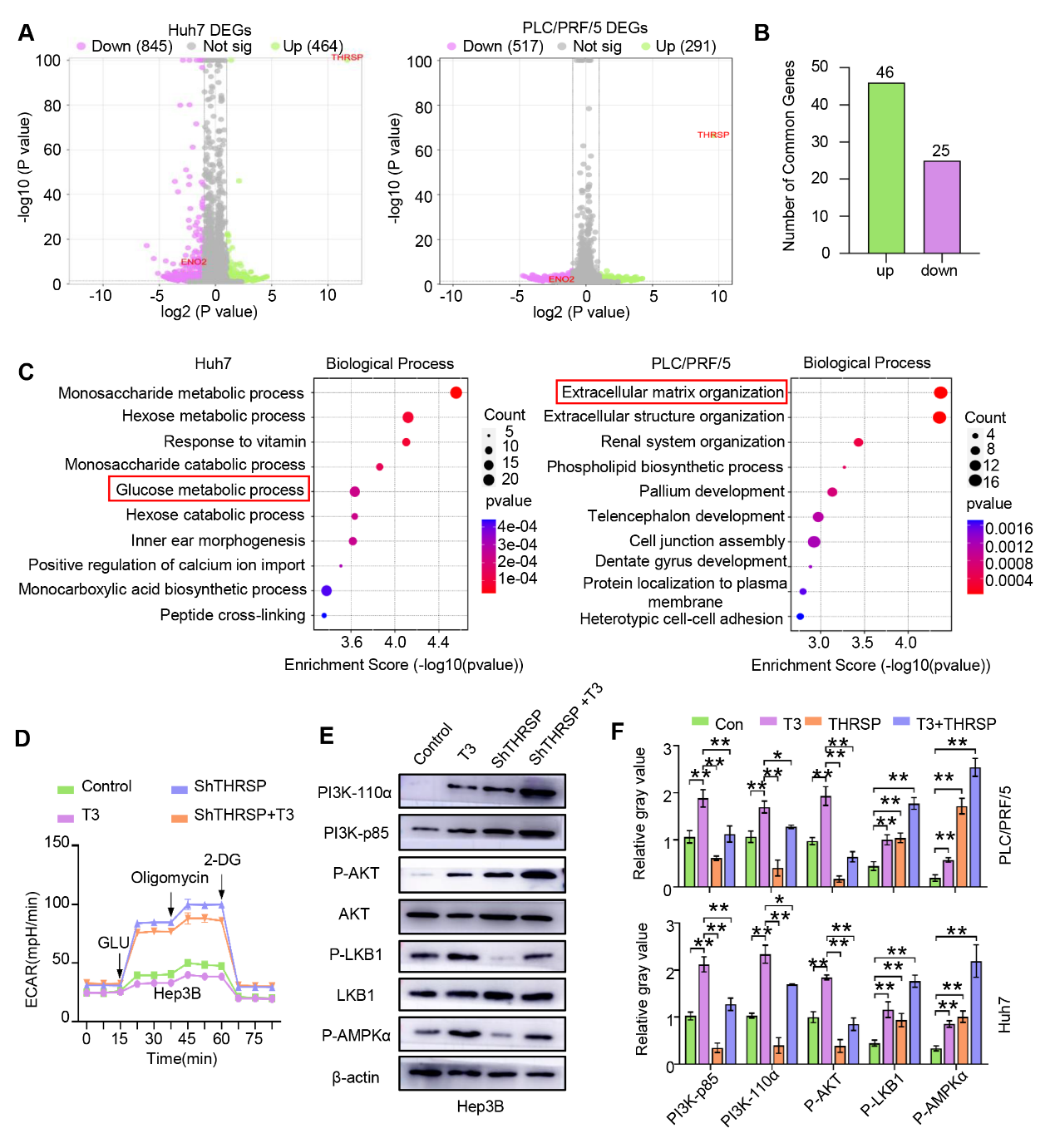
 (A) Volcano plot of DEGs from RNA-seq data. (B) The number of up-regulated and down-regulated genes in DEGs. (C) GO analysis of cellular pathways involved in THRSP-OE HCC cells. (D) Knockdown of THRSP affects the effects of T3 on glycolysis in Hep3B cells. (E) WB revealed that the knockdown of THRSP inhibited the LKB1/AMPK signaling pathway and activated the PI3K/AKT signaling pathway in Hep3B cells. (F) Grayscale scanning analysis of key indicators of PI3K/AKT and LKB1/AMPK signaling pathways. *, P < 0.05; **, P < 0.01.

**Figure S3 THRSP mediates THs-induced HCC inhibition and glucose metabolism by inhibiting the transcription and expression of ENO2**


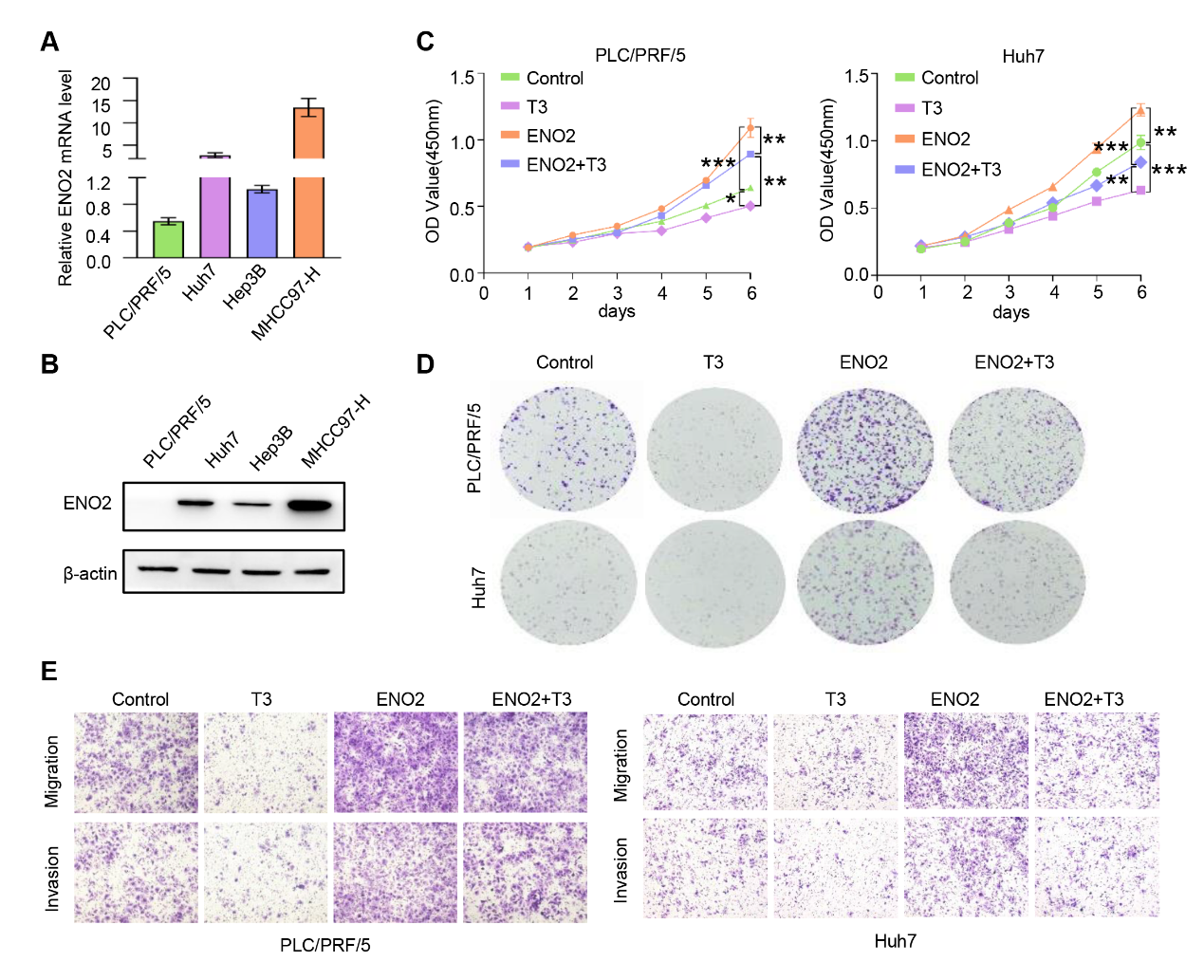
 (A-B) qRT-PCR and WB were used to detect basal ENO2 expression in PLC/PRF/5, Huh7, Hep3B and MHCC97H cell lines. (C-E) Overexpression of ENO2 could attenuate the inhibitory effects of THs on the proliferation, clone formation, migration, and invasion ability of HCC cells. *, P < 0.05; **, P < 0.01; ***, P < 0.001.

**Figure S4 THRSP mediates THs-induced HCC inhibition through inhibiting mTOR- induced HIF-1α nuclear translocation**


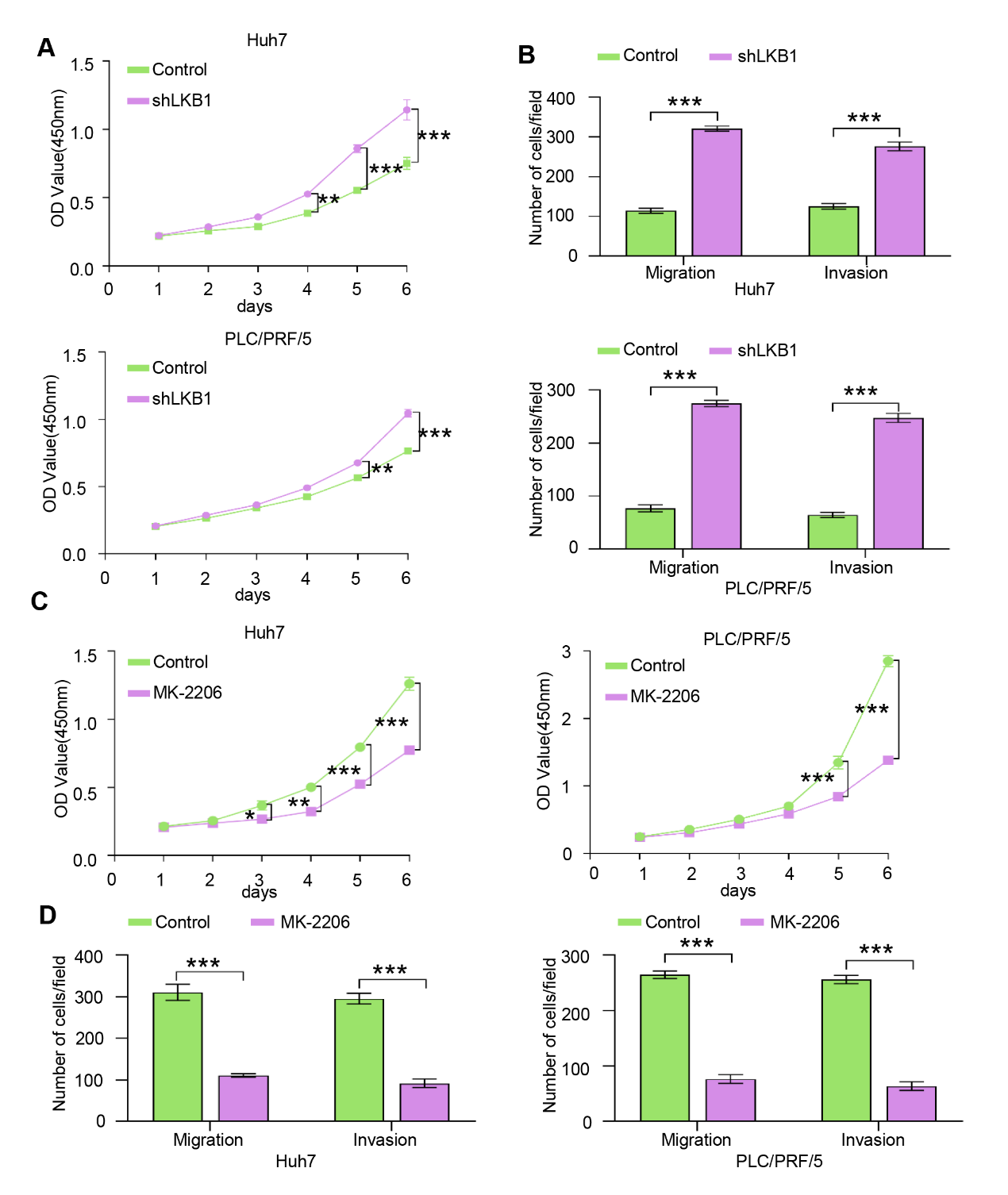
 (A-B) CCK-8 and transwell assays were conducted to determine the effects of LKB1-KD on the cell proliferation, migration, and invasion ability of THRSP-OE cells. (C-D) MK-2206 could further inhibit the proliferation, migration, and invasion of THRSP-OE cells. *, P < 0.05; **, P < 0.01; ***, P < 0.001.

**Figure S5 T3 synergistically enhances the anti-tumor activity of lenvatinib in HCC**


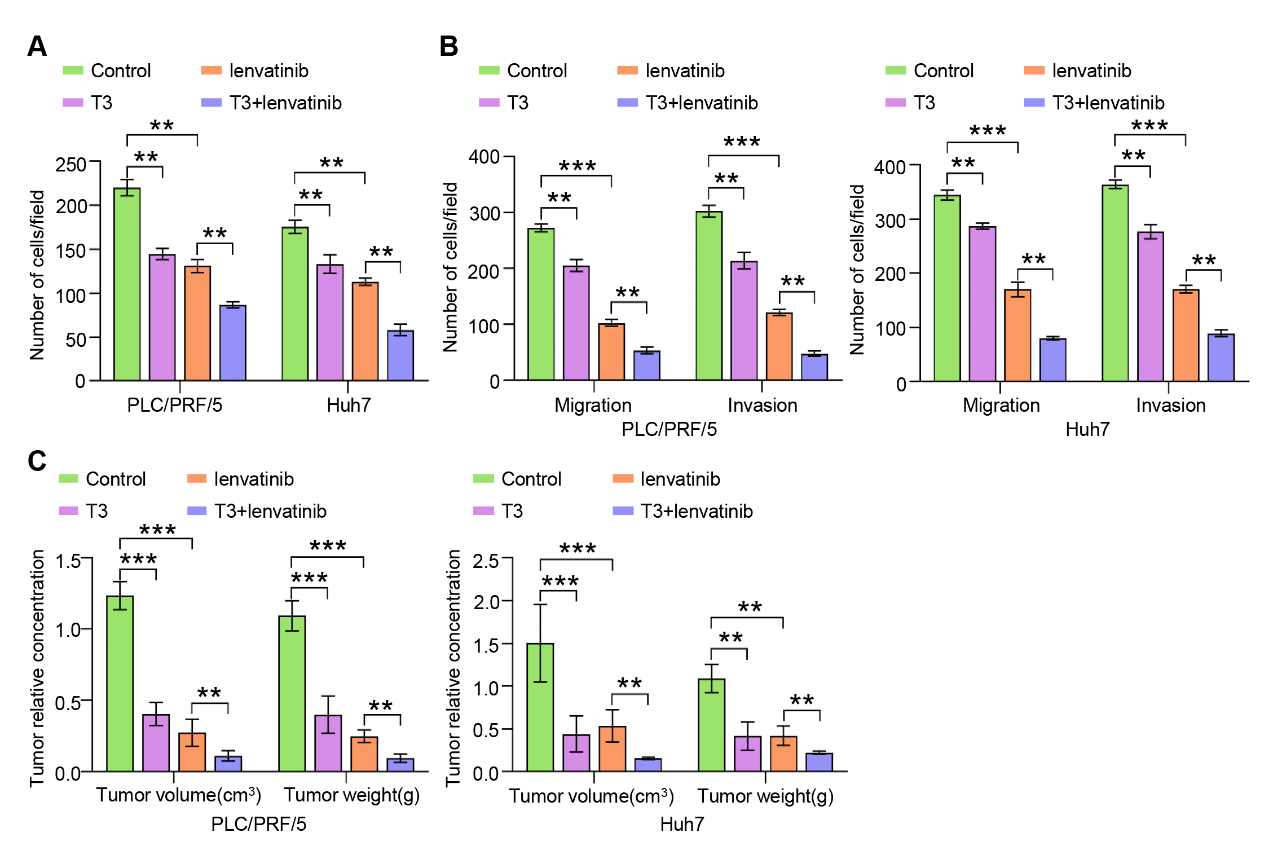
 (A-B) Statistical analysis of cell colony formation and cell numbers in the corresponding groups. (C) Statistical analysis of xenograft tumor volume and tumor weight in the corresponding groups. **, P < 0.01; ***, P < 0.001.

**Supplementary Tables:**

**Table S1. Sequence of gene primers**

| Gene | Forward primer | Reversed primer |
| --- | --- | --- |
| β-actin | GAAGAGCTACGAGCTGCCTGA | CAGACAGCACTGTGTTGGCG |
| THRSP | AACCAAGCGTTACCCCAAGA | ACCACCTGCTCCATGTTGTG |
| ENO2 | TCCCACTGATCCTTCCCGAT | CAGCCCAATCATCCTGGTCA |

**Table S2. Predicted binding information for HIF-1a and human THRSP promoter**

| Matrix ID | Name | Score | Relative score |
| --- | --- | --- | --- |
| MA1106.1 | MA1106.1.HIF1A | 7.785 | 0.87543982748 |
| MA0259.1 | MA0259.1.ARNT::HIF1A | 7.491 | 0.88940569618 |
| MA0259.1 | MA0259.1.ARNT::HIF1A | 7.357 | 0.88540838146 |
| MA1106.1 | MA1106.1.HIF1A | 5.754 | 0.82761569601 |
| MA0259.1 | MA0259.1.ARNT::HIF1A | 5.703 | 0.83606724540 |
| MA0259.1 | MA0259.1.ARNT::HIF1A | 5.615 | 0.83342188118 |
| MA0259.1 | MA0259.1.ARNT::HIF1A | 5.072 | 0.81723553191 |
| MA0259.1 | MA0259.1.ARNT::HIF1A | 4.846 | 0.81049822682 |
| MA0259.1 | MA0259.1.ARNT::HIF1A | 4.798 | 0.80906581849 |
| MA0259.1 | MA0259.1.ARNT::HIF1A | 4.579 | 0.80252910320 |

**Table S3. Univariate and multivariate analysis of OS in HCC patients**

| Variable | Univariate | | Multivariate | |
| --- | --- | --- | --- | --- |
|  | χ2 | *p* value | HR (95%Cl) | *p* value |
| THRSP level (high vs low) | 0.380 | <0.001 | 0.222-0.579 | <0.001 |
| AFP (ng/ml) (≤400 vs >400) | 1.367 | 0.233 | - | n.a. |
| Vascular invasion (yes vs no) | 3.484 | 0.042 | 1.464-13.230 | 0.008 |
| Tumor capsule (none vs yes) | 1.102 | 0.686 | - | n.a |
| Tumor number (≥2 vs 1) | 0.754 | 0.319 | - | n.a |
| Sex (male vs female) | 0.711 | 0.293 | - | n.a. |
| Age (years) (≤50 vs >50) | 1.261 | 0.354 | - | n.a. |
| HBsAg (positive vs negative) | 1.441 | 0.244 | - | n.a. |
| Tumor differentiation (III-IV vs I-II) | 0.754 | 0.319 | - | n.a. |
| Tumor size (≤5cm vs >5cm) | 1.149 | 0.610 | - | n.a. |
| TNM (III-IV vs I-II) | 1.879 | 0.027 | 1.462-2.777 | 0.008 |
| Liver cirrhosis (yes vs none) | 2.576 | 0.014 | 1.337-5.431 | 0.006 |

**Table S4. Univariate and multivariate analysis of RFS in HCC patients**

| Variable | Univariate | | Multivariate | |
| --- | --- | --- | --- | --- |
|  | χ2 | *p* value | HR (95%Cl) | *p* value |
| THRSP level (high vs low) | 0.581 | 0.013 | 0.394-0.890 | 0.012 |
| AFP (ng/ml) (≤400 vs >400) | 0.812 | 0.346 | - | n.a. |
| Vascular invasion (yes vs no) | 1.256 | 0.731 | - | n.a. |
| Tumor capsule (none vs yes) | 1.084 | 0.715 | - | n.a |
| Tumor number (≥2 vs 1) | 1.262 | 0.532 | - | n.a |
| Sex (male vs female) | 0.756 | 0.329 | - | n.a. |
| Age (years) (≤50 vs >50) | 1.187 | 0.450 | - | n.a. |
| HBsAg (positive vs negative) | 1.360 | 0.295 | - | n.a. |
| Tumor differentiation (III-IV vs I-II) | 0.929 | 0.761 | - | n.a. |
| Tumor size (≤5cm vs >5cm) | 0.909 | 0.706 | - | n.a. |
| TNM (III-IV vs I-II) | 1.885 | 0.019 | 1.455-2.532 | <0.000 |
| Liver cirrhosis (yes vs none) | 1.684 | 0.096 | - | n.a. |

**Table S5. Prediction of transcription factor HIF-1α**

| Matrix ID | Name | Score | Relative score | Sequence ID | Start | End | Predicted sequence |
| --- | --- | --- | --- | --- | --- | --- | --- |
| [MA1106.1](https://jaspar.genereg.net/matrix/MA1106.1) | HIF-1α | 12.91 | 0.996 | ENO2 | 1760 | 1769 | GTACGTGCG |
| [MA1106.1](https://jaspar.genereg.net/matrix/MA1106.1) | HIF-1α | 5.42 | 0.820 | ENO2 | 2027 | 2086 | GCACCTGCT |
| [MA1106.1](https://jaspar.genereg.net/matrix/MA1106.1) | HIF-1α | 5.24 | 0.816 | ENO2 | 202 | 211 | GAACGGGCT |
| [MA1106.1](https://jaspar.genereg.net/matrix/MA1106.1) | HIF-1α | 4.69 | 0.802 | ENO2 | 72 | 81 | AGACCTGCC |

**Table S6. Sequence of ENO2 promoter primers**

| ENO2 | Forward primer | Reversed primer |
| --- | --- | --- |
| Primer pair 1 | CATGAACCCCTCCTGACCACA | GGTTATCAAGCAGCACCAACG |
| Primer pair 2 | CTGGTGCAGATGCCATACCT | TTCAGCCTCTGTCGAATCACAT |
| Primer pair 3  Primer pair 4 | CTGCCTGTTACTGTCGAGTGG  CCTCTCCGCATCTCTGGC | CCCCCGATGAGTCAGGAGC  AGAAAGCGAAGGCAGCGG |

**Table S7. Univariate and multivariate analysis of OS in HCC patients**

| Variable | Univariate | | Multivariate | |
| --- | --- | --- | --- | --- |
|  | χ2 | *p* value | HR (95%Cl) | *p* value |
| ENO2 level (high vs low) | 4.121 | <0.001 | 2.586-7.002 | <0.001 |
| AFP (ng/ml) (≤400 vs >400) | 1.242 | 0.421 | - | n.a. |
| Vascular invasion (yes vs no) | 2.124 | 0.206 | - | n.a. |
| Tumor capsule (none vs yes) | 1.175 | 0.505 | - | n.a |
| Tumor number (≥2 vs 1) | 1.095 | 0.825 | - | n.a |
| Sex (male vs female) | 0.876 | 0.686 | - | n.a. |
| Age (years) (≤50 vs >50) | 1.378 | 0.194 | - | n.a. |
| HBsAg (positive vs negative) | 1.557 | 0.167 | - | n.a. |
| Tumor differentiation (III-IV vs I-II) | 0.714 | 0.245 | - | n.a. |
| Tumor size (≤5cm vs >5cm) | 0.986 | 0.958 | - | n.a. |
| TNM (III-IV vs I-II) | 2.456 | 0.003 | 1.832-3.484 | <0.001 |
| Liver cirrhosis (yes vs none) | 2.397 | 0.027 | 1.164-4.705 | 0.017 |

**Table S8. Univariate and multivariate analysis of RFS in HCC patients**

| Variable | Univariate | | Multivariate | |
| --- | --- | --- | --- | --- |
|  | χ2 | *p* value | HR (95%Cl) | *p* value |
| ENO2 level (high vs low) | 1.574 | 0.036 | 1.017-2.240 | 0.041 |
| AFP (ng/ml) (≤400 vs >400) | 0.800 | 0.319 | - | n.a. |
| Vascular invasion (yes vs no) | 1.181 | 0.795 | - | n.a. |
| Tumor capsule (none vs yes) | 1.073 | 0.750 | - | n.a |
| Tumor number (≥2 vs 1) | 1.252 | 0.547 | - | n.a |
| Sex (male vs female) | 0.821 | 0.497 | - | n.a. |
| Age (years) (≤50 vs >50) | 1.205 | 0.410 | - | n.a. |
| HBsAg (positive vs negative) | 1.287 | 0.387 | - | n.a. |
| Tumor differentiation (III-IV vs I-II) | 0.873 | 0.577 | - | n.a. |
| Tumor size (≤5cm vs >5cm) | 0.833 | 0.469 | - | n.a. |
| TNM (III-IV vs I-II) | 1.956 | 0.015 | 1.436-2.498 | <0.000 |
| Liver cirrhosis (yes vs none) | 1.661 | 0.107 | - | n.a. |
