# Supplementary material for: Thyroid hormones inhibit tumor progression and enhance the antitumor activity of lenvatinib in hepatocellular carcinoma via reprogramming glucose metabolism
Source: Cell Death Discov. 2025 Mar 8;11:92. doi: 10.1038/s41420-025-02378-z (PMC11889155; doi:10.1038/s41420-025-02378-z)
Supplement: Supplementary file 2 — Gel [file 41420_2025_2378_MOESM2_ESM.pptx]

## Slide 1
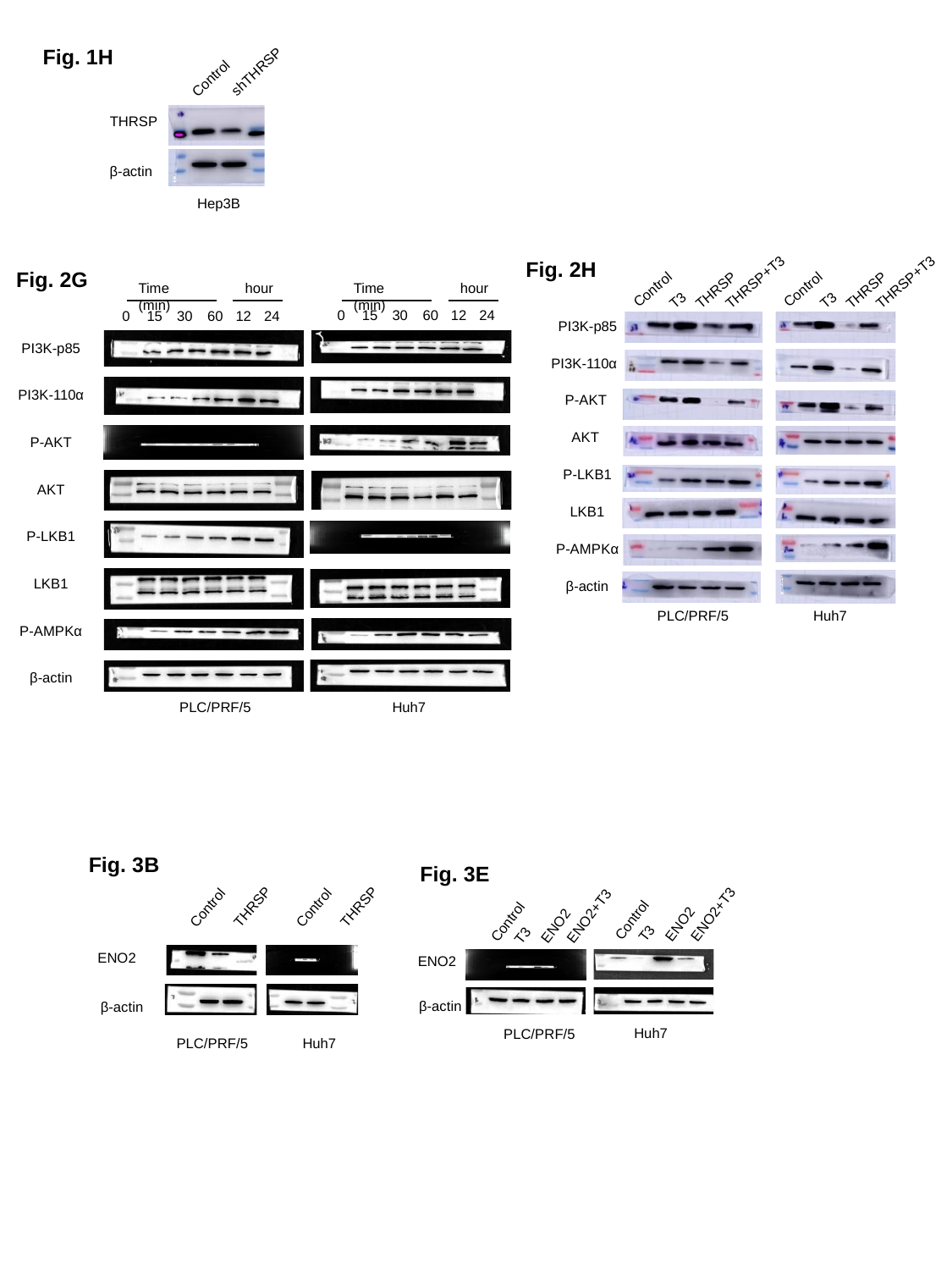

Fig. 1H
shTHRSP
Control
THRSP
β-actin
Hep3B
Fig. 2H
Control
Control
THRSP
THRSP+T3
THRSP
THRSP+T3
T3
T3
PI3K-p85
PI3K-110α
P-AKT
AKT
P-LKB1
LKB1
P-AMPKα
β-actin
PLC/PRF/5
Huh7
Fig. 2G
hour
Time (min)
hour
Time (min)
0
15
30
60
12
24
0
15
30
60
12
24
PI3K-p85
PI3K-110α
P-AKT
AKT
P-LKB1
LKB1
P-AMPKα
β-actin
PLC/PRF/5
Huh7
T3
ENO2+T3
ENO2
Control
ENO2
β-actin
Huh7
PLC/PRF/5
Fig. 3B
Fig. 3E
THRSP
THRSP
Control
Control
ENO2
β-actin
PLC/PRF/5
Huh7
T3
ENO2+T3
ENO2
Control

## Slide 2
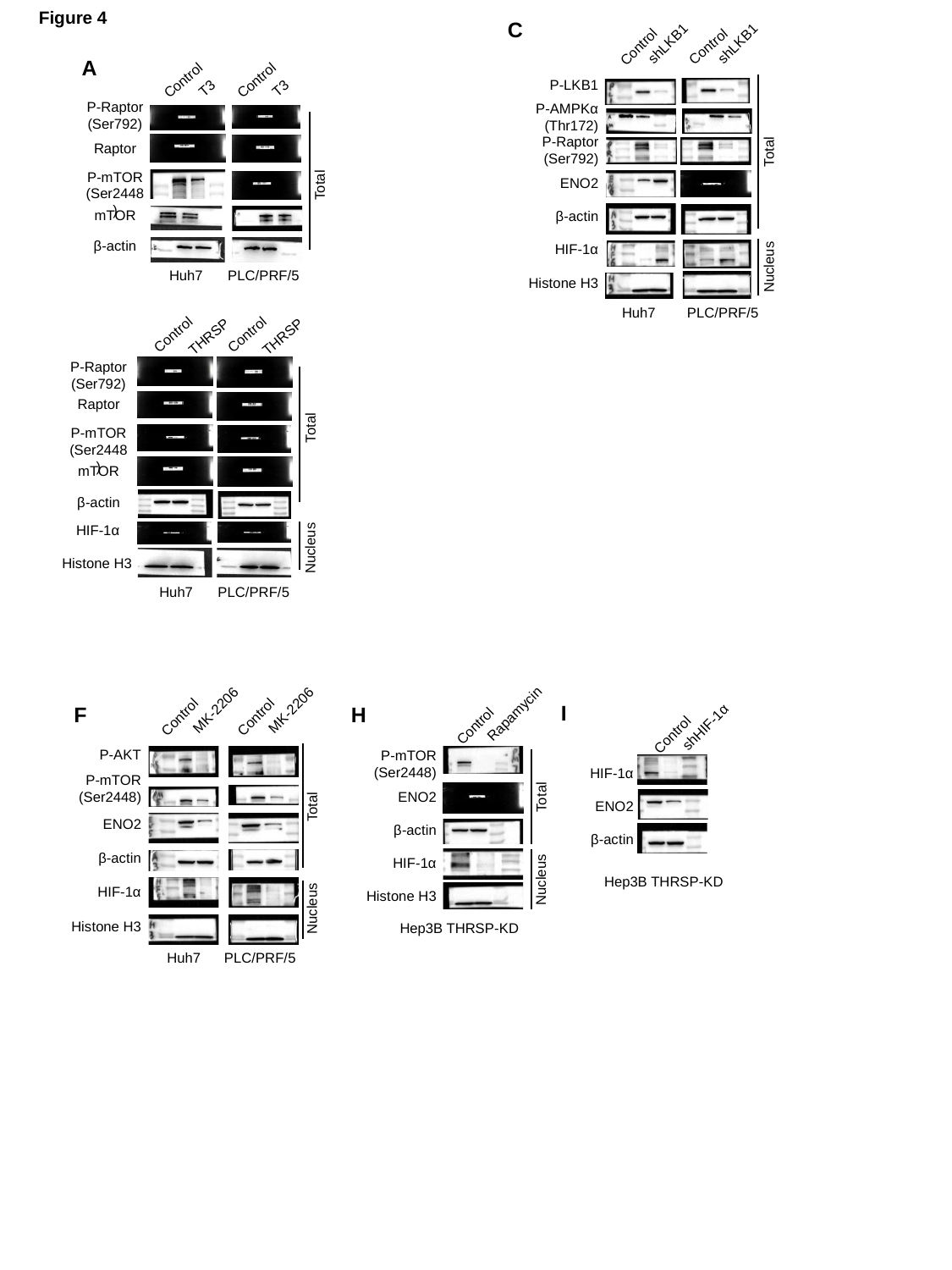

Figure 4
C
shLKB1
shLKB1
Control
Control
P-LKB1
P-AMPKα
(Thr172)
P-Raptor
(Ser792)
Total
ENO2
β-actin
HIF-1α
Nucleus
Histone H3
PLC/PRF/5
Huh7
A
Control
Control
T3
T3
P-Raptor
(Ser792)
Raptor
P-mTOR
(Ser2448)
mTOR
β-actin
Huh7
PLC/PRF/5
Total
Control
Control
THRSP
THRSP
P-Raptor
(Ser792)
Raptor
P-mTOR
(Ser2448)
mTOR
β-actin
HIF-1α
Histone H3
Huh7
PLC/PRF/5
Total
Nucleus
MK-2206
MK-2206
Control
Control
P-AKT
 P-mTOR
(Ser2448)
Total
ENO2
β-actin
HIF-1α
Nucleus
Histone H3
PLC/PRF/5
Huh7
I
F
H
Rapamycin
Control
P-mTOR
(Ser2448)
ENO2
β-actin
HIF-1α
Histone H3
Hep3B THRSP-KD
Total
Nucleus
Control
shHIF-1α
HIF-1α
ENO2
β-actin
Hep3B THRSP-KD

## Slide 3
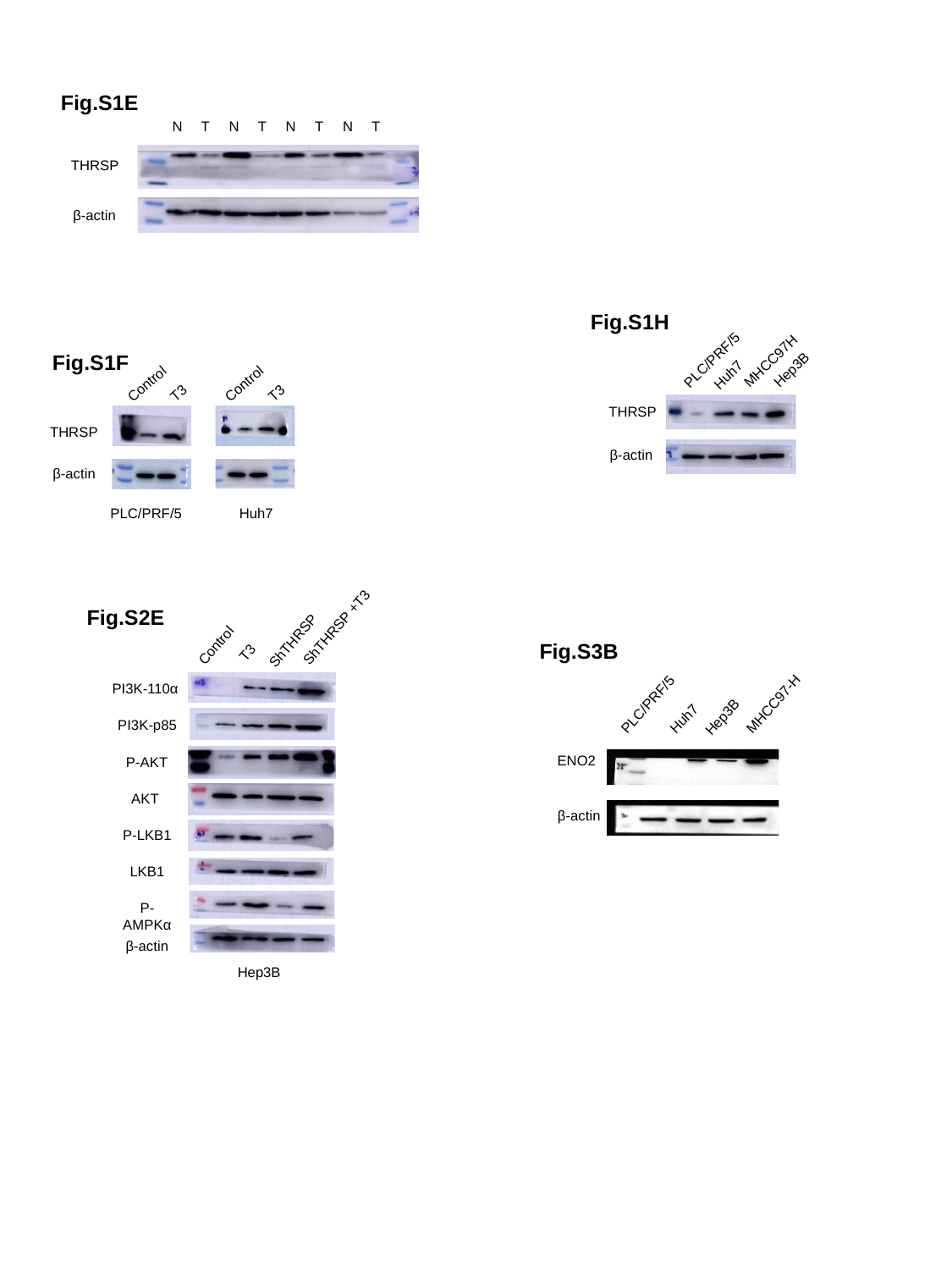

Fig.S1E
N
T
N
T
N
T
N
T
THRSP
β-actin
Fig.S1H
PLC/PRF/5
Hep3B
MHCC97H
Huh7
THRSP
β-actin
Fig.S1F
Control
T3
Control
T3
THRSP
β-actin
PLC/PRF/5
Huh7
T3
ShTHRSP
Control
PI3K-110α
PI3K-p85
P-AKT
AKT
P-LKB1
LKB1
P-AMPKα
β-actin
Hep3B
ShTHRSP +T3
Fig.S2E
PLC/PRF/5
MHCC97-H
Hep3B
Huh7
ENO2
β-actin
Fig.S3B
